# Supplementary figures and images for: Enhanced Removal of Endocrine-Disrupting Compounds from Wastewater Using Reverse Osmosis Membrane with Titania Nanotube-Constructed Nanochannels
Source: Membranes (Basel). 2022 Sep 30;12(10):958. doi: 10.3390/membranes12100958 (PMC9609337; doi:10.3390/membranes12100958)

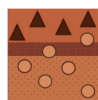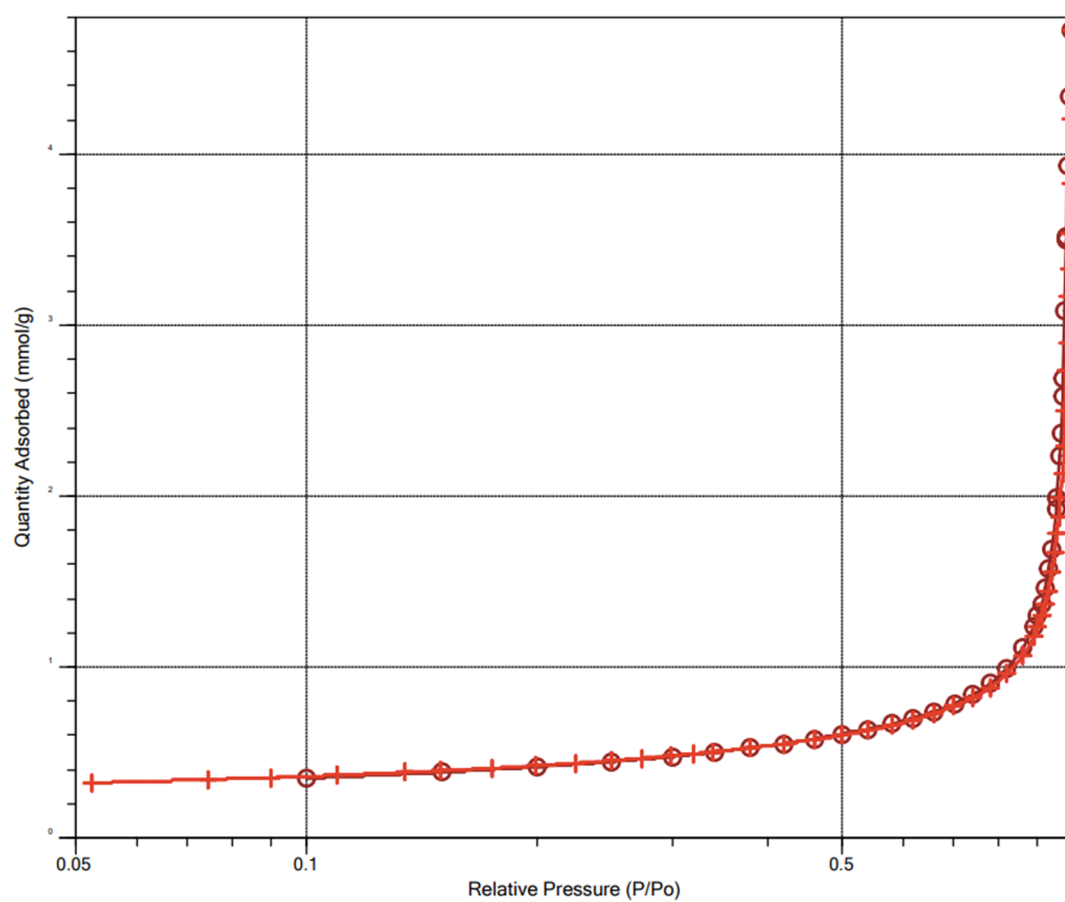

**Figure S1.** N<sub>2</sub> adsorption-desorption isotherms.

Supplement: Supplementary file 1 [file membranes-12-00958-s001.zip › membranes-1930593-supplementary.pdf]
